# Description of a clinical decision support tool with integrated dose calculator for paediatrics

**DOI:** 10.1007/s00431-021-04261-2

**Published:** 2021-09-15

**Authors:** Lukas Higi, Karin Käser, Monika Wälti, Michael Grotzer, Priska Vonbach

**Affiliations:** 1 PEDeus Ltd, Zurich, Switzerland; 2 Department of Pharmaceutical Sciences, University of Basel, Basel, Switzerland; 3 University Children’s Hospital of Zurich, Zurich, Switzerland

**Keywords:** Clinical decision support, Paediatric, Dose calculation, Medication error, Paediatric dosing, Patient safety

## Abstract

Medication errors, especially dosing errors are a leading cause of preventable harm in paediatric patients. The paediatric patient population is particularly vulnerable to dosing errors due to immaturity of metabolising organs and developmental changes. Moreover, the lack of clinical trial data or suitable drug forms, and the need for weight-based dosing, does not simplify drug dosing in paediatric or neonatal patients. Consequently, paediatric pharmacotherapy often requires unlicensed and off-label use including manipulation of adult dosage forms. In practice, this results in the need to calculate individual dosages which in turn increases the likelihood of dosing errors. In the age of digitalisation, clinical decision support (CDS) tools can support healthcare professionals in their daily work. CDS tools are currently amongst the gold standards in reducing preventable errors. In this publication, we describe the development and core functionalities of the CDS tool *PEDeDose*, a Class IIa medical device software certified according to the European Medical Device Regulation. The CDS tool provides a drug dosing formulary with an integrated calculator to determine individual dosages for paediatric, neonatal, and preterm patients. Even a technical interface is part of the CDS tool to facilitate integration into primary systems. This enables the support of the paediatrician directly during the prescribing process without changing the user interface.

*Conclusion*: *PEDeDose* is a state-of-the-art CDS tool for individualised paediatric drug dosing that includes a certified calculator.

**Table:** 

**What is Known:** *• Dosing errors are the most common type of medication errors in paediatric patients.* *• Clinical decision support tools can reduce medication errors effectively. Integration into the practitioner’s workflow improves usability and user acceptance.*	
**What is New:** *• A clinical decision support tool with a certified integrated dosing calculator for paediatric drug dosing.* *• The tool was designed to facilitate integration into clinical information systems to directly support the prescribing process. Any clinical information system available can interoperate with the PEDeDose web service.*

**What is Known:**

*• Dosing errors are the most common type of medication errors in paediatric patients.*

*• Clinical decision support tools can reduce medication errors effectively. Integration into the practitioner’s workflow improves usability and user acceptance.*

**What is New:**

*• A clinical decision support tool with a certified integrated dosing calculator for paediatric drug dosing.*

*• The tool was designed to facilitate integration into clinical information systems to directly support the prescribing process. Any clinical information system available can interoperate with the PEDeDose web service.*

## Introduction

In 2018, the World Health Organisation (WHO) addressed the high burden of medication errors (ME) by initiating the third Global Patient Safety Challenge: *Medication without harm* [1]. ME are preventable errors that arise due to inappropriate medication use by healthcare professionals (HCP) or patients [2]. Compared to adults, the paediatric population is considered to be affected by ME more frequently [3–6]. Besides that, paediatric patients are also exposed to a high proportion of unlicensed and off-label prescribed drugs [7–9]. Furthermore, the lack of an appropriate dosage or a suitable galenic form for paediatric patients often requires manipulation of the product by nurses and pharmacists, such as grinding tablets or diluting liquids [10]. Additionally, clinical trials for regulatory approval are not often done in the paediatric population [11, 12]. Extrapolation of dosage data from different age groups is feasible; however, it remains a challenging issue as the paediatric patient population is very heterogenous due to a multitude of developmental changes [13–15]. These include changes in body composition, maturation of organs, or age-dependent variation in the activity of metabolising enzymes [13, 16]. Not only the dosage itself but also the conversion to the dispensing unit is a well-reported source of errors [17, 18]. Thus, the leading causes of preventable errors in paediatric patients are prescribing errors, particularly dosing errors [3, 5, 19].

Even though it is now over 20 years after the US Institute of Medicines report *To err is human* [20] was published, effective reduction of ME is still an ongoing issue [21]. Nowadays, the widespread use of clinical information systems (CIS) and computerised provider order entry systems (CPOE) allow for the implementation of innovative digital health tools such as clinical decision support (CDS) systems to assist HCP in their daily work. Nevertheless, the introduction of digital health tools also brings new challenges and potential sources for errors [6]. Moreover, it was shown that the introduction of a CPOE without CDS was not effective in reducing ME [22–24]; however, the usability of CDS tools is depending on the quality and ease of integration [6, 24, 25]. Currently, there are not many CDS available that target paediatric medication safety and allow for integration into a primary system [6].

The term medical device covers a heterogenous group of tools including software such as CDS systems. In the recent years, several failures of medical devices certified under the European medical device directive (MDD) were reported [26–28]. With the new European medical device regulation (MDR) coming into effect, superseding the former MDD, a stricter regulatory framework is defined [29]. Thus, with the expiry of the transition period (end of May 2021) most digital health tools will be classified into a higher risk class under the new MDR. Compliance with regulatory standards, which includes numerous explicitly mentioned norms and guidelines, is an extensive and time-consuming process, but above all it contributes to the safety of medical devices. As for a digital health tool that is aimed at reducing preventable errors this is of utmost importance — *primum non nocere*.

In this publication, we describe the CDS tool *PEDeDose* (https://www.pededose.ch/en/) [30], which is certified as a medical device Class IIa according to the MDR. PEDeDose provides evidence-based individualised dosage information for HCP treating paediatric or neonatal patients. Our tool enables HCP to search, verify, and calculate individualised drug dosages according to the provided evidence. The aim of PEDeDose is to prevent incorrect dosages already during the prescribing process and to reduce the workload of the clinical pharmacists validating dosages. Additional information about PEDeDose can be obtained on the website of the company (https://www.pedeus.ch/en/). This publication emphasises on the development, the management of the dosing data, and the clinical applicability of PEDeDose.

## Development and certification

PEDeDose was launched in 2019 by PEDeus, a subsidiary company of the University Children’s Hospital of Zurich. As a legal manufacturer the quality management system of PEDeus was built adhering to ISO 13485:2016 [31] and to the European MDR [29]. Our product PEDeDose was built in close collaboration with the University Children’s Hospital of Zurich. PEDeDose consists of three main modules: website, web service, and data management. The website provides a user interface, which is compatible with the most frequently used internet browsers. The web service allows the integration of PEDeDose into primary systems such as a CIS, medical practice, or pharmacy software and enables the use of additional features. The web service exposes a state-of-the art Representational State Transfer (REST) Application Programming Interface (API) for the primary system to consume using JavaScript Object Notation (JSON). The data management module was developed for maintenance of the dosing database and to ensure traceability and archiving. It is only used by our clinical experts that are trained to add, review, or edit the dosage data. The software was developed using open-source systems, namely the PHP framework Symfony [32] and the relational database MariaDB [33].

PEDeDose was developed in compliance with the MDR [29] according to IEC 62304 [34], which defines the development of medical device software. The software behind PEDeDose was classified according to the Medical Device Coordination Group (MDCG) guidance [35] and the evaluation of the International Medical Device Regulators Forum (IMDRF) [36]. The following two main principles were considered: (1) the impact of the information that is provided to the HCP and (2) the current situation in healthcare. The final decision, whether to use the information provided by PEDeDose, remains with the HCP and needs to be actively accepted by the HCP. Thus, PEDeDose was considered a medical device software that “informs clinical management”, but since PEDeDose is intended for the fragile paediatric population the healthcare situation was considered as “critical”. PEDeDose does not achieve its functionality by direct action on the human body, which in turn reduces the patient’s risk. The result of this evaluation leads to the certification of the software behind PEDeDose as a Class IIa medical device according to the MDR [29].

## Clinical application

**Fig. 1:**
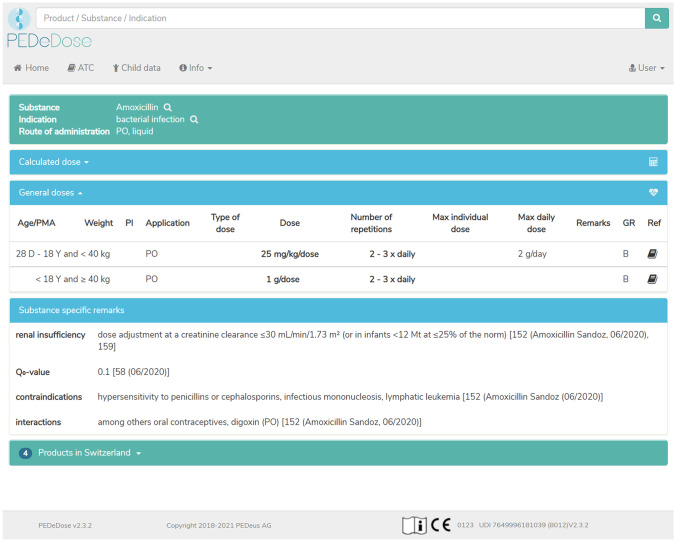
Screenshot of PEDeDose: general information and substance specific remarks for a liquid oral formulation of the antibiotic amoxicillin

**Fig. 2:**
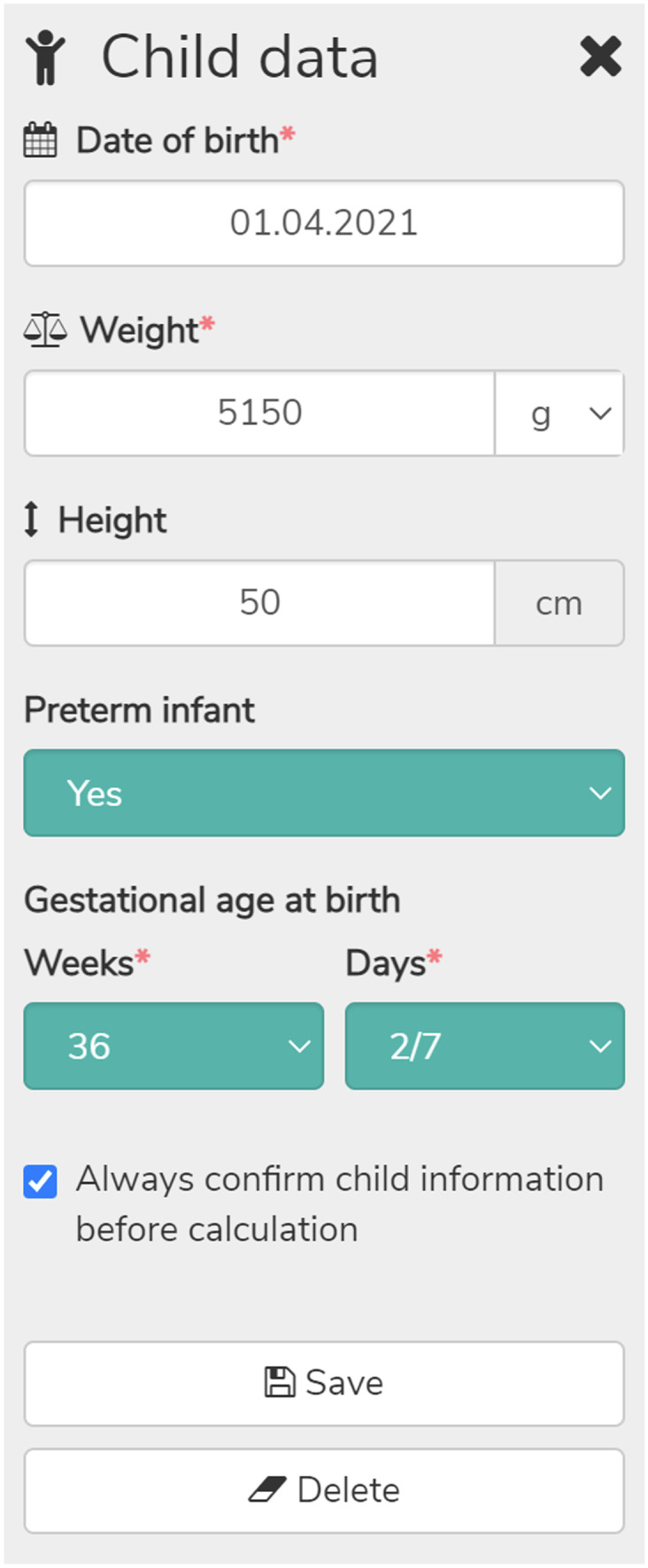
Screenshot of the PEDeDose child data entry form. The data for a hypothetical preterm child with a body weight of 5150 g was entered

**Fig. 3:**
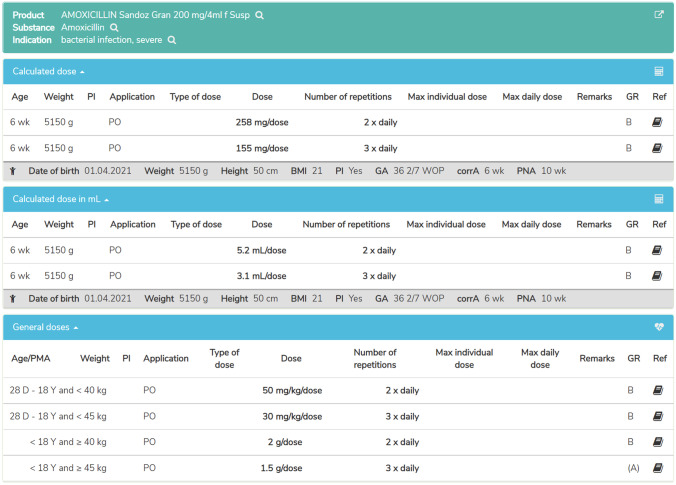
Screenshot of the individualised dosage calculation for a liquid oral amoxicillin formulation for the specified child data. For this example, a hypothetical preterm child of 5150-g weight was selected

The target audience of PEDeDose comprises all HCP working with paediatric patients including neonates and preterm infants, thus comprising not only hospitals, but also physician practices and public pharmacies. PEDeDose can be used to search for active ingredients, products, indications, or by the Anatomical Therapeutic Chemical (ATC) classification system. Upon selection of a product or active ingredient, an indication, a route of administration, and a galenic form the general dosing information is presented. Alongside each dosing information, the data source and the grade of recommendation are provided. Additional substance specific remarks, for example the need for dose adjustment in case of renal or liver insufficiency, drug-drug interactions, or undesirable effects, are provided as well. Figure 1 shows the general information provided by PEDeDose. An individualised dosing calculation can be made by entering the child’s age, weight, height (optionally), and birth status (preterm: yes/no). In case of a preterm infant the gestational age at birth will be asked as well. If there is a need to calculate several dosages for the same patients, the user must specifically tick the checkbox to keep the entered child’s data. This is a safety measure to prevent carrying over the first patient’s data to the next. The child data entry form is shown in Fig. 2. Additionally, as a special feature currently limited for Swiss users, PEDeDose displays products available on the Swiss market. Upon selection of a specific product for a calculated dosage, PEDeDose will convert the individual dose into dispensing units according to the galenic form and the administration aid of the product (e.g. milligrams to drops or millilitres) (Fig. 3). However, the database is built around the active ingredient and the products are then added to the corresponding active ingredient. Thus, one can always search for active ingredients only, but the possibility remains to add products of various countries to the active ingredients in the future.

PEDeDose is already in use in all the three German-speaking Swiss Children’s Hospitals and further paediatric clinics, as well as in several local public pharmacies and medical practices. The tool is currently available in three different languages: English, French, and German. Due to its responsive design the software can adapt to the different screen sizes (desktop, tablet, or mobile) that may be used in these different environments. To this day, our database contains information on 321 active ingredients and 1896 products with market authorisation in Switzerland.

## Interoperability and integration into primary systems

**Fig. 4:**
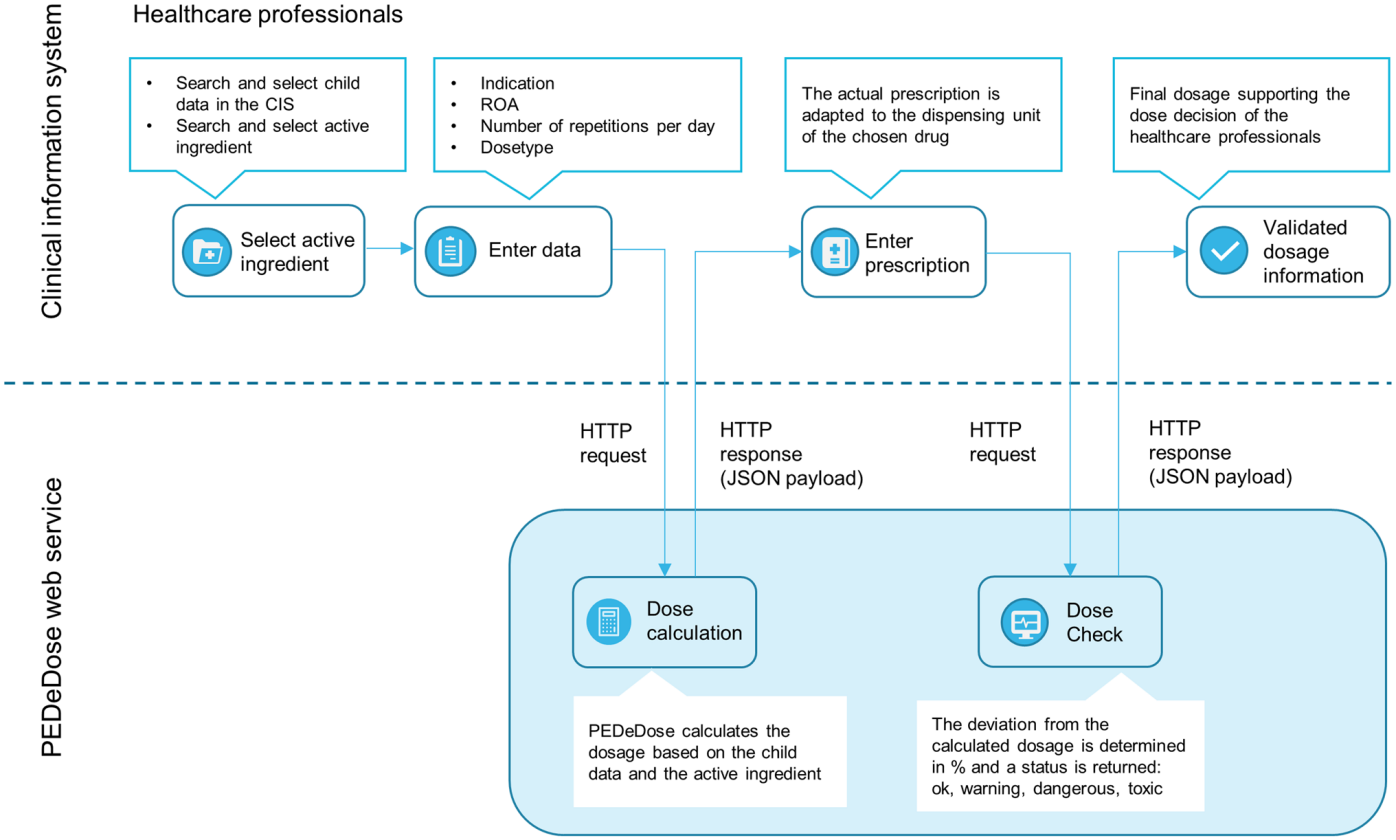
A schematic overview of the interoperation between the clinical information system (CIS) and the PEDeDose web service. Starting with a dose request by a healthcare professional (HCP) that is sent to PEDeDose. Then PEDeDose returns the individual dosage to the CIS. After the HCP enters the prescription, the dosage may be checked again by the dose check functionality

There are two main ways to use PEDeDose, either via its website using an internet browser or via its web service. The advantage of the web service lies within the possibility to support the prescribing process comprehensively. PEDeDose can then be accessed directly via the user interface of the primary system, without the need to switch to the PEDeDose website. The primary system (e.g. a CIS) can then interoperate with the PEDeDose web service; this means it can forward the necessary child parameters (e.g. weight, birth date) to PEDeDose. In return the PEDeDose web service sends back the individual dosage, which is then displayed by the CIS (Fig. 4). The advantages of a web service to provide a CIS with specific paediatric functionalities, such as a CDS, was even recently acknowledged by a policy statement of the American Academy of Pediatrics [37]. Since nowadays most hospitals use electronic ordering and prescribing processes, it is imperative to integrate the CDS into the CIS, as only then the full potential of the CDS can be exploited. Currently, this includes providing individualised dosages and automated checking of the prescribed dosages for correctness.

The automated dose check provides the HCP with the deviation from the recommended dosage in percent, together with an alert indicating the level of the deviation. For each active ingredient, it is defined whether there is a broad or narrow therapeutic range. The therapeutic range in turn defines to which extent the dosage may deviate from the recommendation, which is 10% and 5% for ingredients with broad and narrow therapeutic range, respectively, for the first level of alert.

Analysis of the *PEDeDose* log data showed that the use of the PEDeDose calculator at the University Children’s Hospital of Zurich, a 211-bed hospital [38], increased enormously after integration into the CIS. The substantial increase in calculator use highlights the importance of developing CDS tools that can be integrated into primary systems.

## Data management

To ensure a high quality of the dosing data, a data management module with its own interface was developed. This allowed us to design safety measures directly into the software. In the data management all actions performed by its operators are logged, thus allowing to trace back modifications during the data update process. There are specific roles assigned to each operator that allow to control the workflow. Editors may add or edit a dataset, whilst the reviewer is not allowed to manipulate the data. Once an editor adds or edits a new dataset, two different reviewers (clinical experts) will revise the dosage dataset independently (six-eye principle). During an ongoing reviewing process, the editor will be blocked from editing the dataset, thus preventing that unverified or manipulated data is published.

The dosages provided by PEDeDose are determined in a standardised procedure. A defined dosage dataset is always linked to a specific active ingredient, indication, route of administration, and galenic form. To prevent the publication of overlapping dosing information (age, weight, indication, route of administration, etc.) the data management module automatically checks newly entered data with the already available data and will prohibit the recording of overlapping data.

To enter a new dosage into the database, the following steps are considered: (1) *literature search*: this includes searching primary literature databases (e.g. Pubmed, Cochrane Library, Embase), specialised literature (e.g. RedBook by the American Association of Pediatrics), or published information by regulatory authorities (e.g. Swissmedic, European Medicines Agency). The literature is rated according to the strength of evidence into grades of recommendation [39] (2) *SwissPedDose database*: the SwissPedDose database contains harmonised dosage information [40], is supported by all eight Swiss Children’s hospitals, and financed by the government. The harmonisation process is based on a consensus-driven approach which generally leads to a Swiss-wide acceptance of the dosage data. (3) *Discussion with experts*: based on the previous search protocol the proposed dosages are discussed with clinical experts (the experts are mainly chief physicians or pharmacists of the University Children’s Hospital of Zurich). The final dosage is determined by synthesising the results of said procedure, aiming at the highest grade of recommendation. Thus, if a dosage is already approved by regulatory authorities it is integrated without further appraisal. However, as the levels of evidence decrease, the extent of the literature and database search increases and a final discussion with experts is required.

Dosing information is preferably defined as fixed dosages and not dose ranges. Whenever possible the maximum dose is provided in the same unit as the recommended dose to prevent misinterpretation. Decimal values below 0.01 are changed to the next unit (e.g. from milligram to microgram).

Topicality of our database is ensured by periodically reviewing novel scientific publications, literature alerts, or newsletters published by health authorities. Sporadically or annually published documents, such as vaccination plans and antidote lists are reviewed upon release. Direct exchange with the Swiss University Children’s hospitals or other defined children’s hospitals ensures clinical appropriateness of the provided data.

## Safety measurements

The development of a medical device requires an initial and continuous assessment and evaluation of potential risks including strategies to prevent or minimise potential harm. The risk management process was done in accordance with ISO 14971:2016 [41] as required by the MDR. We identified the following four main hazards: (1) *Overdose*, (2) *Wrong medication*, (3) *Delayed prescription of medication*, and (4) *Breach of privacy*. For each hazard potential risks were identified, whilst for each risk control measures were defined and implemented. To this day, a total of 58 different risks were identified and 77 unique risk control measures were implemented. Table 1 shows a selection of potential risks identified and the respective control measures.

**Table 1:** Selection of potential risks, their implications for the users, and the corresponding control measures in PEDeDose

**Hazardous situation**	**Hazard type**	**Implications**	**Risk control measure**
Incomplete dosage data input in the data management	Delayed prescription of medication	Dosage row cannot be found or used anymore, therefore no calculation of dosage possible	Data entries are checked regarding consistency Modified entries are directly visible in the release process Mandatory release process according to the six-eye-principle Access restricted to HCP
Unintended input/confused term for gathering child data	Overdose	Dosage is calculated based on incorrect child data	The child input form must be explicitly requested for user input. Each child data input field must be explicitly selected. Dedicated control for date entry (calendar), gestational age, weight unit, pre-term infant Data entries are checked regarding consistency Access restricted to HCP Calculation of dosage is only available if valid child information was provided After successful calculation the website shows the calculated dosages, the child information used for the calculation, and percentile check warnings Before saving, child information is checked on plausibility using the percentile check. If warnings exist, they have to be confirmed.
Service and maintenance operations lead to downtime due to problems or unfavourable maintenance time	Delayed prescription of medication	PEDeDose not usable, no calculation of dosages possible	Installation requires a pre-defined approval and validation Update requires a pre-defined approval and validation Local installation
Unauthorised people get access to personal information	Breach of privacy	Personal information protection is compromised	Access restricted to HCP User’s personal data is not transferred to the local installation Software does not use nor collect sensitive personal patient data

A top priority of PEDeDose is the around the clock availability, as relying on a CDS entails an increased dependency on it. Unavailability of the tool may result in delayed prescription of medication, which can lead to serious harm or even death of a patient [42]. Thus, risk control measures include pre-defined approval and validation prior to software updates as well as protection measures against cyberattacks. To ensure around the clock availability PEDeDose is hosted using a fully redundant system and may additionally be installed locally in a hospital. Updates of dosing data are immediately synchronised, also with the local installation. Another important risk poses the management of the dosing data as incorrect or incomplete data may result in the prescription of a wrong dose. Thus, a data management module was developed using a safety by design approach, as described before. The dosing calculator was validated using automated unit testing, which verified software requirements at the code level. Validity of calculator functionality was verified by automated integrated testing and additional manual testing. Logging data of the dosing calculator are systematically collected. The calculator log data collection is validated, so potential erroneous calculations can be fully backtracked.

The safety of a medical device software must not only be looked at from a technical point of view, but includes human factors as well; thus the usability of the software has to be assessed repeatedly upon changes in the web application. Upon a new release the usability of PEDeDose is tested by HCP from diverse professional areas and variable experience with CDS or information technologies. The usability test requires the HCP to solve test calculations using PEDeDose on three different devices (PC, tablet, smartphone). To this day the usability tests confirmed excellent usability and the intuitive use of PEDeDose even without prior consultation of the instruction manual.

Furthermore, the MDR [29] requires implementation of post-market surveillance, including user feedback, and continuous market observation for product and supplier related information. The post-market surveillance also considers problems and safety signals of comparator products.

## Comparison with other paediatric CDS tools

To the best of our knowledge, there are only few published CDS tools for paediatric dosing available that are either certified and/or include a broad variety of active ingredients or products [6]. Thus, we want to highlight the website *Kinderformularium.nl*, which is based on the Dutch Paediatric Formulary [43]. Their comprehensive database provides high-quality information on more than 750 drugs [43]. Additionally, van der Zanden et al. evaluated the impact of their tool in a simulation study showing that the use of their dosing calculator led to a 15% reduction of overall calculation errors [44]. However, their calculator is currently disabled since November 2020. The calculator is notified according to the MDD as medical device of Class I. There are some other online dosing calculators available, such as the iDoseCalc [45], neda.io [46], and the IBM Micromedex [47]. However, very limited or even no information about the development and validation of these tools or on certification or notification according to MDR or MDD, respectively, is published.

## Strengths and limitations

PEDeDose provides HCP with easily accessible evidence-based and individualised dosing recommendations. The use of PEDeDose is not only aimed at reducing errors but can also save precious time in case of emergency situations. One of the main strengths of our CDS is the dosing calculator that is already certified under the stricter requirements of the new MDR. We are not aware of other comparable certified paediatric CDS tools that are currently available in Europe. In the literature, a main point of critique of CDS lies within poor interoperability with the institution’s existing infrastructure [17, 48]. PEDeDose, however, can be integrated via web service, which additionally allows for customisation of its interface based on the institution’s needs. The integration of PEDeDose via web service allows for the direct prescription of drugs via CPOE. Dose checking and unit conversion are two functionalities that Gildon et al*.* argued should be standard for CDS [17]. PEDeDose provides both of those functionalities.

Another general point of critique of digital health tools is the loss of user skill whilst relying on the tool [49]. However, this problem lies within the nature of a CDS; thus we approached this by ensuring around the clock availability using redundant servers and the possibility for a local installation. Even though our goal is to improve paediatric patient safety around the globe, certain additional features such as the link to commercially available products are currently tailored for Swiss users, as PEDeDose was initially developed within a Swiss children’s hospital.

## Conclusion

Dosing errors are a leading cause of preventable harm in paediatric patients. The current literature describes CDS as a promising measure to reduce preventable errors. With the introduction of the MDR CDS must comply with high regulatory standards to ensure the patient’s safety. In this publication, we describe the CDS tool *PEDeDose*, a Class IIa medical device software certified according the MDR. A web service allows to integrate the software into a primary system, fulfilling a main point of concern of the literature — the interoperability. The CDS provides evidence-based dosing information and an integrated dosing calculator to determine individualised dosages. The aim of the tool is to support HCP treating paediatric and neonatal patients enabling them to make informed evidence-based decisions. Studies evaluating the efficacy and efficiency of PEDeDose will be needed to explicitly quantify its clinical impact.

## Abbreviations

API: Application programming interface
ATC: Anatomical Therapeutic Chemical Classification System
CDS: Clinical decision support
CIS: Clinical information system
CPOE: Computerised provider order entry system
HCP: Healthcare professionals
IMDRF: International Medical Device Regulators Forum
JSON: JavaScript Object Notation
MDCG: Medical Device Coordination Group
MDD: Medical Device Directive
MDR: Medical Device Regulation
ME: Medication errors
REST: Representational state transfer
WHO: World Health Organisation
